# Low-temperature flow-synthesis-assisted urethane-grafted zinc oxide-based dental composites: physical, mechanical, and antibacterial responses

**DOI:** 10.1007/s10856-021-06560-4

**Published:** 2021-07-28

**Authors:** Jaffar Hussain Bukhari, Abdul Samad Khan, Kashif Ijaz, Shahreen Zahid, Aqif Anwar Chaudhry, Muhammad Kaleem

**Affiliations:** 1 Department of Dental Materials, Army Medical College, National University of Medical Sciences, Rawalpindi, 46000 Pakistan; 2 Department of Dental Materials, Nishtar Institute of Dentistry, Nishtar Medical University, Multan, 64000 Pakistan; 3 Department of Restorative Dental Sciences, College of Dentistry, Imam Abdulrahman Bin Faisal University, Dammam, 31441 Saudi Arabia; 4 Interdisciplinary Research Centre in Biomedical Materials, COMSATS University Islamabad, Lahore Campus, Lahore, 54000 Pakistan; 5 Department of Dental Materials, Shifa Medical and Dental College, Islamabad, 44000 Pakistan

## Abstract

A novel way was adopted to graft zinc oxide (ZnO) with urethane-modified dimethacrylate (UDMA) in order to utilize them as reinforcing agents in resin-based dental composites. Experimental novel composites were synthesized having UDMA-grafted and nongrafted ZnO, at a concentration of 0 wt.%, 5 wt.%, and 10 wt.%. The same concentrations of ZnO were also incorporated in Filtek Z250 XT (3 M ESPE, USA). The antibacterial behavior was evaluated against Streptococcus *mutans* by direct-contact test at one, three, and seven days of incubation. The compressive strength and Vickers microhardness were tested as per ISO 9917 and ISO/CD6507-1, respectively. For abrasive wear resistance, mass loss and roughness average after tooth-brushing cycles of 24,000 at custom-made tooth-brushing simulator were evaluated using noncontact profilometer. Data analysis was carried out using post hoc Tucky’s test and nonparametric Kruskal–Wallis test. Direct contact test revealed that the antibacterial potential of novel and commercial composites was increased with an increase in the concentration of grafted ZnO as compared with nongrafted, whereby the potential was the highest at day seven. There was a significant decrease in compressive strength and Vickers hardness of commercial composites on addition of grafted ZnO while there was no significant difference in the strength of experimental novel composite. The abrasive wear of commercial and experimental composites was within clinical limits. Low-temperature flow-synthesis method was successfully employed to synthesize grafted and nongrafted ZnO. The UDMA-grafted ZnO can be incorporated into dental composites without decreasing their strength and these composites can be used to combat secondary caries.

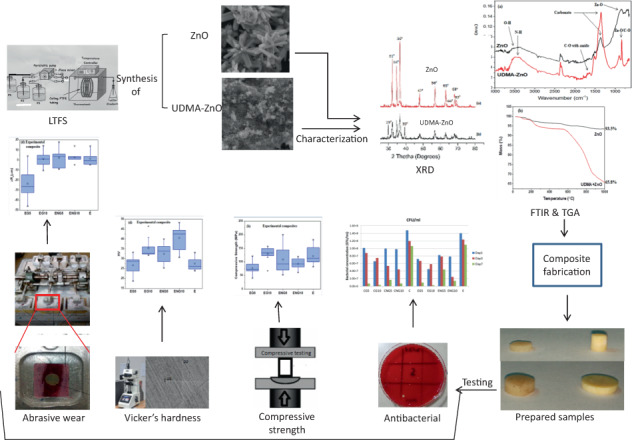

## Introduction

It is desirable for current dental restorative materials to have antibacterial properties, as the occurrence of plaque accumulation on resin-based composites (RBCs) is higher, subsequently, leading to secondary caries formation [1, 2]. One of the methods to make the RBCs resistant to oral bacterial accumulation is addition of antibacterial agents, mainly chlorhexidine, fluoride, and benzalkonium chloride [3–5]. However, the issue with these antibacterial agents is that after sometime, their effect diminishes, subsequently there is an adverse effect on the mechanical properties of the restoration [6, 7]. Another approach is the inclusion of quaternary ammonium that inactivates only the bacteria coming in direct contact with active molecules that are immobilized or incorporated as the functional group. Therefore, the inactivating effect does not reach the areas around the dental composite restoration [8, 9]. Other promising agents are metallic nanoparticles, which due to increased surface area, increased longevity, and inherent properties exhibit better chemical and bactericidal activity [10]. However, silver and titanium oxide (TiO_2_) nanoparticles had shown discoloration [11] and decreased shear bond strength with increasing the concentration of TiO_2_ [12].

Among these metallic nanoparticles, zinc oxide (ZnO) showed effectiveness against cariogenic bacteria; moreover, nanoparticles exhibited better performance compared with larger particles [13, 14]. ZnO nanoparticles have a tendency to get agglomerated and show low dispersion ability in the polymeric network, therefore, their application is largely limited. To overcome this limitation, the grafting of ZnO particles with polymers has been employed [15].

The techniques for the fabrication of ZnO nanoparticles are vaporization, micro emulsion synthesis [16], spray drying [17], sol–gel method [18], pyrolysis [19], controlled precipitation [20], and solution phase [21]. However, synthesis by these methods at the large scale is not cost-effective. Recently, flow-synthesis reactors have been used for the production of nanoparticles of metal oxides [22–24]. In such reactors that employ flow chemistry, the resultant product (particles) is uniform with high reproducibility due to the homogeneous mass of the reacting components and controlled temperature in the zone where the reaction takes place [23].

The grafting or silanization of dental fillers is a determining factor related to the physical and mechanical properties of resin-based composites. Whereby, 3-methacryloxypropyl trimethoxysilane (MPS) has been used commonly, however, grafting of ZnO with MPS increased the diameters of the aggregates, even to the order of micrometers [25]. During the silanization process, several factors can affect coupling efficiency, such as the nature of solvent, pH, and concentration of the coupling agent [26]. Whereby, increased silane can lead to a detrimental effect on the properties of the material [27]. The conventional way of stirring for a period of 8–48 h has been used to silanize filler particles with γ-methacryloxypropyltrimethoxysilane [28]. Therefore, in this study, a novel in situ technique, i.e., flow-synthesis method was used to graft particles in the shortest possible time. The aim was to utilize flow-synthesis method to graft ZnO particles with urethane dimethacrylate (UDMA). It is anticipated that UDMA can make a direct bond with resin matrix system and minimize the problems associated with interfaces between fillers and resins. It is hypothesized that the UDMA–ZnO particles would show stability and improved antibacterial activity without decreasing the mechanical properties.

## Materials and methods

### Synthesis of nongrafted and grafted ZnO particles

All precursors used for the synthesis of zinc oxide particles (ZnO) were of analytical grade and were purchased from Sigma-Aldrich, USA. Zinc oxide particles were synthesized in hydrothermal reactor. The separate solutions of 0.3 M zinc nitrate and 0.6 M sodium hydroxide were prepared with deionized water. Both solutions were stirred at room temperature for 15–20 min separately. Low-temperature flow synthesis (LTFS) was switched on and clean water was run before the experiment. The heater was switched on and set at a temperature, such that the exiting water temperature was ~70 °C. The flow rate of both solutions was set at ~45 mL/min and both the solutions were pumped simultaneously. The exit temperature was 50 °C and product solution was filtered and oven-dried (Model OFA-54-8, ESCO, Singapore) at 40 °C for 24 h. For grafted ZnO, the same procedure was followed, except the addition of UDMA solution prepared with ethanol and exit temperature was 40 °C. The three solutions were pumped through a peristaltic pump using three streams at a flow rate of ~ 45 mL/min and an x-piece mixer was used after the pump. The obtained product solution was filtered and oven-dried at 40 °C for 24 h.

### Synthesis of the experimental composite

For the synthesis of the experimental novel composite, bisphenol-A glycerolate dimethacrylate (*bis*-GMA), triethylene glycol dimethacrylate (TEGDM), and diurethane dimethacrylate (UDMA) were supplied by Sigma-Aldrich, USA. Camphorquinone (CQ) and ethyle 4-dimethylaminobenzoate (EDMAB) were supplied by Alpha Aesar, Germany, and silicon dioxide by Dalian Richon chemical Co, China. Before the synthesis of the experimental novel composite, silica was silanized with MPS by a reported method [29]. To synthesize the experimental novel composite, a measured quantity of monomers (*bis*-GMA, TEGDMA, and UDMA having the ratio 40:25:35) was mixed for 30 min on a stirrer in the dark environment at room temperature. The prepared grafted and nongrafted ZnO were incrementally added in the resin matrix, whereby the concentrations were 0, 5, and 10% wt/wt. The mixture was allowed to stir at room temperature (23 ± 2 °C) for 1 h to get a homogeneous mixture. Later, CQ (0.2 wt.%) and EDMAB (0.15 wt.%) were added sequentially. Silanized silica (40 wt.%) was added in increments and stirred at 320 rpm for 2 h to get homogeneous mixing of the ingredients. The mixture was kept in a convection oven at 35 °C for 24 h.

To evaluate the effect of the incorporation of grafted and nongrafted ZnO into the commercial composite, Filtek™ Z250 XT (3 M ESPE, St Paul, USA, A2 shade) was used in the study. The measured quantity of Z250 was slightly plasticized with 0.5 mL of ethanol, then grafted and nongrafted ZnO nanoparticles (5 and 10% wt/wt) were dissolved in Z250, respectively, and mixed at room temperature with the help of a stainless-steel spatula till the evaporation of ethanol. The compositional groups of commercial and experimental composites are given in Table 1.

**Table 1:** Composition and groups of composite used in the current study

Commercial composite (Filtek Z250 XT)			
Group name	Organic components	Inorganic fillers	
		Reinforcing Agents	Active ingredient
CG5	*bis*-GMA, *bis*-EMA, UDMA, and TEGDMA	Silanized Zirconia,/ Silica 82% (w/w)	UDMA grafted ZnO, 5 wt.%
CG10			UDMA grafted ZnO, 10 wt.%
CNG5			Non-grafted ZnO, 5 wt.%
CNG10			Non-grafted ZnO, 10 wt.%
C			as received from the manufacturer
**Experimental composite**			
EG5	*bis-*GMA, TEGDMA, and UDMA	Silica 40% (w/w)	UDMA grafted ZnO, 5 wt.%
EG10			UDMA grafted ZnO, 10 wt.%
ENG5			Non-grafted ZnO, 5 wt.%
ENG10			Non-grafted ZnO, 10 wt.%
E			0 wt.%

### Characterization

#### Grafted and nongrafter zinc oxide

To characterize the UDMA-grafted and nongrafted ZnO, Fourier transform infrared (FTIR) spectroscopy, thermogravimetric analysis (TGA), and X-ray diffraction (XRD) were employed. FTIR spectra were obtained using Nicolet 6700 FTIR, Thermo Scientific, USA. The method employed for spectral analysis was attenuated total reflectance (ATR) mode with a scan range of 400–4000 cm^−1^ at a scan speed of 256 and 8 cm^−1^ resolutions.

The weight loss percent of both nongrafted and grafted ZnO was evaluated by thermogravimetric analysis using TGA Q600, TA Instruments, USA. The grafting percentage of UDMA-grafted ZnO particles was evaluated by the equation as described previously (Kaur et al., 2013)

1 $$\% \,grafting = \left( {W_0 - W_1/W_1} \right)100$$

where, W_0_ = weight of the grafted ZnO before heating and W_1_ = weight of ZnO after removal of the grafting agent.

XRD pattern was obtained by XPERT-PRO diffractometer, PANalytical UK, using Cu K_*α*_ radiation of wavelength *λ*=0.1541 nm in the scan range of 2*θ* angles from 5° to 90° at a step size of 0.02°.

#### Experimental composite

##### Antibacterial testing

The antibacterial activity of all the composite groups was determined by direct-contact test. A total of 30 circular disks (4 × 1 mm) were fabricated and cured with LED-curing light (COXO DB-686 Latte, Guangdong, China) having a wavelength of 420–480 nm and power intensity of 1200 mW/cm^2^. Antibacterial activity was observed periodically at one, three, and seven days with Streptococcus *mutans* (ATCC 25175, ATCC, Manassas, VA, USA). The bacterial suspension in brain–heart infusion (BHI) broth with a concentration of 0.5 McFarland was prepared (1 mL of solution contains approximately 1.5 × 10^8^ bacteria). A visual method was used to count the bacterial colonies. In order to decrease the number of bacterial colonies and make it easy to count them, 0.5 McFarland suspension was diluted 1000 times to achieve a concentration of 1.5 × 10^5^ bacteria in 1 mL. A sampler was used to place 0.005 mL of the bacterial suspension on the surface of the disc samples already sterilized by immersion in 70% ethanol solution for 30 min. Then the samples containing bacterial suspension were placed in presterilized cryogenic vials and were incubated for 1 h in 5% CO_2_ incubator at 37 °C.

After each time interval of incubation (one, three, and seven days), a sterile sampler was used to retrieve 0.005 mL from each liquid culture medium to uniformly spread on a blood agar plate. The blood agar plates were incubated for 48 h under 5% CO_2_ at 37 °C. After the given time, the number of bacterial colonies (colony forming unit, CFU) per plate was visually counted. Bacterial concentration is reported in terms of CFU/mL, whereby the experimental procedure was done in triplicates.

##### Compressive strength

A total of 100 cylindrical specimens (*n* = 10) were prepared according to ISO 9917. The samples were polished and finished with silicon carbide paper in a sequence of 800, 1500, and 2000 grit size. All the samples were stored in deionized water at 37 °C for seven days prior to testing [30]. The samples were blot-dried and transferred to Universal Testing Machine, Testometrics, UK. The force was applied at a crosshead speed of 0.5 mm/min. The stress–strain graph was obtained and the compressive strength (CS) was calculated by the following equation [31]:

2 $${{{{{\mathrm{CS}}}}}} = {{{{{\mathrm{F}}}}}}/\pi {{{{{\mathrm{r}}}}}}^2$$

where, “F” is the load applied and “r” is the half-diameter of the cylinder.

##### Vickers hardness

Vickers hardness was measured according to ISO/CD6507-1 by Digital Microhardness Tester (HVS1000, Sinowon, China). A total of 30 samples, three for each group, were fabricated in a PTFE mold (3 × 8 mm). Three indentations were made on each sample at three different points, with a load of 0.49 N for a dwell time of 15 s. The diagonal D1 and D2 obtained in each indentation were measured microscopically and put in the software for the auto-conversion of Vickers hardness (HV).

##### Abrasive wear testing

Abrasive wear testing was carried out according to IS0/TR 14569. A total of 60 disc-shaped specimens (10 × 2 mm) were prepared (*n* = 6) using a PTFE mold. Samples were thoroughly cured from both sides as mentioned above. Each specimen was conditioned in deionized water for seven days at 37 °C and then air-dried.

Prior to the abrasive test, samples were weighed and the initial mass (M_1_) for each sample was obtained. The initial surface roughness was assessed by using a noncontact mode 2D Profilometer (PS-50 Nanovea, USA), having 2-μm diamond stylus. A measuring length of 8 mm, cut-off length of 0.8 mm, and a stylus speed of 1 mm/s were used. Micrographs were obtained at different scan areas measuring 10 × 10 µm. The roughness data points (Ra, initial values) were taken and means were obtained.

Abrasive wear test was carried out using a custom-made toothbrush simulator constructed according to ISO11609:2010 specifications. It was equipped with six stations of replaceable brush heads (Oral B Flat end). Tooth-brushing load of 1.5 N was set. A commonly used toothpaste (Colgate-Palmolive, Ireland) slurry made with distilled water in the proportion 2:1 was used. The samples were mounted in impression compound and placed in metallic stations. Tooth brushing was accomplished for 24,000 cycles (simulating to three years), at a frequency of 4 Hz with horizontal movements of the tooth brush and a traveled course of 4.2 cm. The slurry was refurbished continuously throughout testing, replaced, and freshly formed for each sample. The brush heads were also replaced.

Following the abrasive wear test, the samples were carefully removed, rinsed in tap water, and placed in an ultrasonic cleaner (VGT1860-QTD, MTI Corporation, USA) for 1 min. The samples were then individually retrieved, air-dried, and weighed. In this way, the final mass (M_2_) was obtained for each sample and post-abrasion percentage mass loss (ΔM) was calculated for each sample by the equation, ΔM = M_1_−M_2_

##### Change in roughness average

The final surface roughness average (Ra_f_) was measured after the abrasive wear test, using the profilometer in the same way as for the initial values, except that the tracing arm of the profilometer was positioned in such a way that the tracing direction was perpendicular to the line of tooth brushing. The change in roughness average (ΔRa) was calculated by the equation: ΔRa = Ra_i_−Ra_f_

### Statistical analysis

IBM Statistical package for social sciences (SPSS) version 22 was used for data analysis. Shapiro–Wilk test was applied to check the normality of data. *p*-value ≤ 0.05 was considered as statistically significant. The data of antibacterial testing followed the normality curve and were analyzed with multivariate ANOVA (CFU, time intervals, and compositional groups). The pairwise comparison (post hoc analysis) was done by using the simple main effect.

The data of compressive strength, Vickers hardness, and roughness average followed the normality curve and were analyzed by univariate ANOVA followed by post hoc Tucky’s test. The data of mass loss were analyzed by nonparametric Kruskal–Wallis test followed by post hoc Tuckey’s test.

## Results

**Fig. 1:**
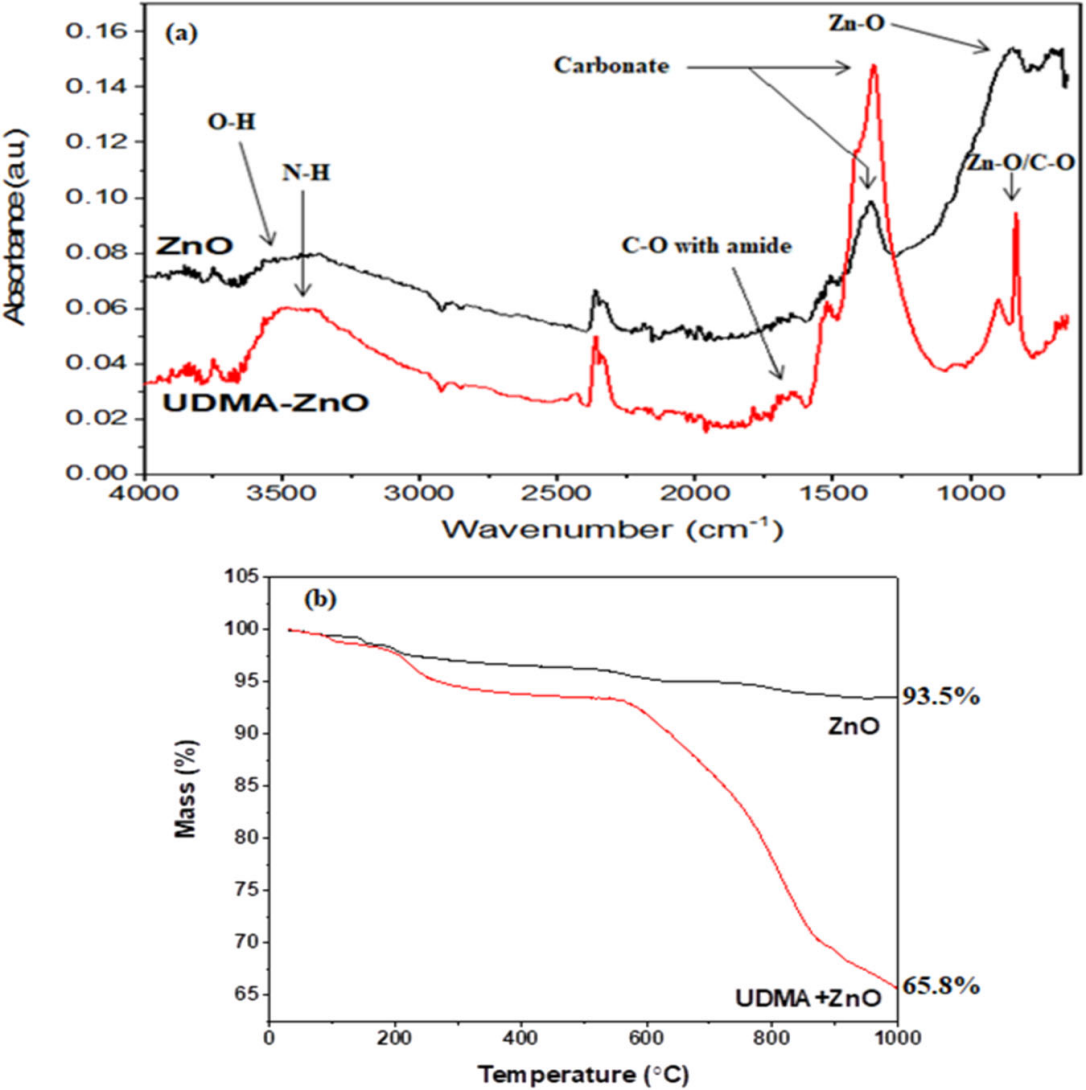
**a** FTIR spectra of UDMA-grafted and nongrafted ZnO particles **b** TGA graph of pure and UDMA-grafted ZnO showing the mass loss after heating at 1000 °C

Figure 1a showed the FTIR absorption spectra of ZnO particles and UDMA-grafted ZnO. The broad band between 3600 and 3000 cm^−1^ attributed to the stretching vibration of the OH group, however, after grafting, the band shape changed to a prominent peak (3450–3350 cm^−1^) assigned to the N–H group due to the presence of UDMA. The sharp peak at 1750–1650 cm^−1^ was assigned to the linkage of stretching vibrations of the C–O group with the amide peak of UDMA. The peaks observed at (ZnO and UDMA–ZnO) 1400–1300 cm^−1^ were attributed to carbonate peak. The peak at 700–800 cm^−1^ was assigned to Zn–O with an additional C–O group.

The TGA thermogram showed (Fig. 1b) the comparative mass loss percentage of grafted and nongrafted ZnO. The nongrafted ZnO showed mass loss after 100 °C, which was due to loss of moisture and impurities. At 1000 °C, the mass loss was 6.5%. The UDMA-grafted ZnO showed mass loss from 90 to 290 °C due to loss of moisture and the initial decomposition of UDMA. Another drastic mass loss was noted at 560 °C, which was due to the final decomposition of UDMA, and at 1000 °C mass loss was 34.2%. This confirmed the presence of UDMA on the surface of ZnO and the total mass loss of UDMA was approximately 28%, which was close to the initial weight percentage of UDMA.

**Fig. 2:**
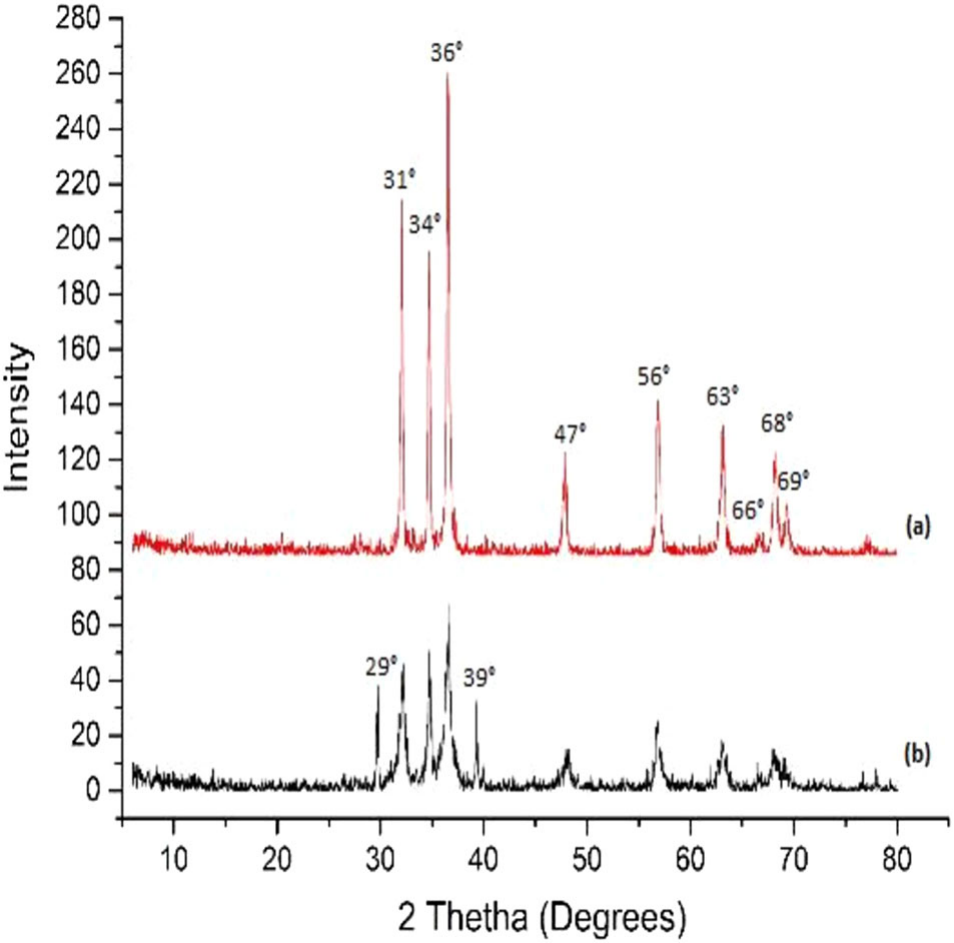
XRD pattern showing peaks of (**a**) pure ZnO and (**b**) UDMA-grafted ZnO

The XRD pattern shown in Fig. 2 (a), verified that peaks exhibited by pure ZnO at 31°, 34°, 36°, 47°, 56°, 63°, 66°, 68°, and 69° are in accordance with reference 00–036, International Centre for Diffraction Data (ICDD). The two additional peaks at 29° and 39° (Fig. 2b) showed the presence of UDMA onto the surface of ZnO particles.

### Antibacterial testing

**Fig. 3:**
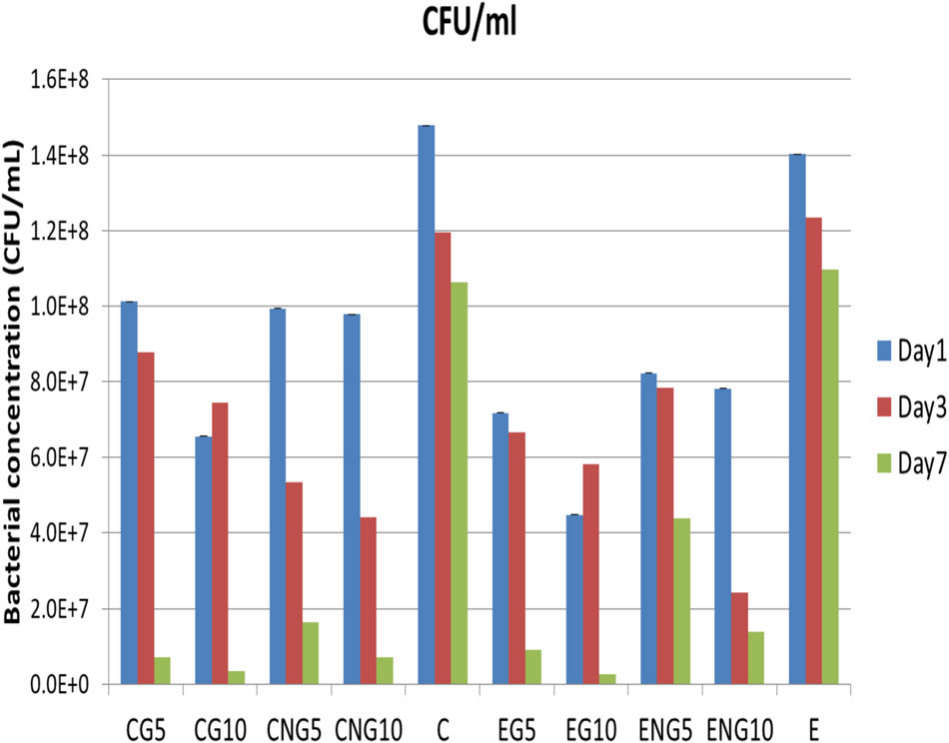
CFU/mL of commercial composite groups and experimental novel composite groups at days one, three, and seven

The mean values of CFU/mL of commercial and experimental composites at days one, three, and seven are given in Fig. 3. The mean CFU/mL values of the experimental groups (EG5, EG10, ENG5, and ENG10) and commercial groups (CG5, CG10, CNG5, and CNG10) were less than the mean CFU/mL of their negative control groups (E) and (C), respectively, at days one, three, and seven. The difference was statistically significant (*p* < 0.05). The nonlinear behavior was observed among groups, whereby both control groups (C and E) showed maximum CFU/mL compared with other groups. The commercial composite groups showed higher values at day one compared with experimental composite groups. The trend showed that CFU/mL values were decreased with time and a significant difference was observed between day 1 and 7, among all groups. At day seven, CFU/mL values of the groups having UDMA-grafted ZnO were significantly lesser (*p* < 0.05) than the groups having nongrafted ZnO.

### Compressive strength

**Fig. 4:**
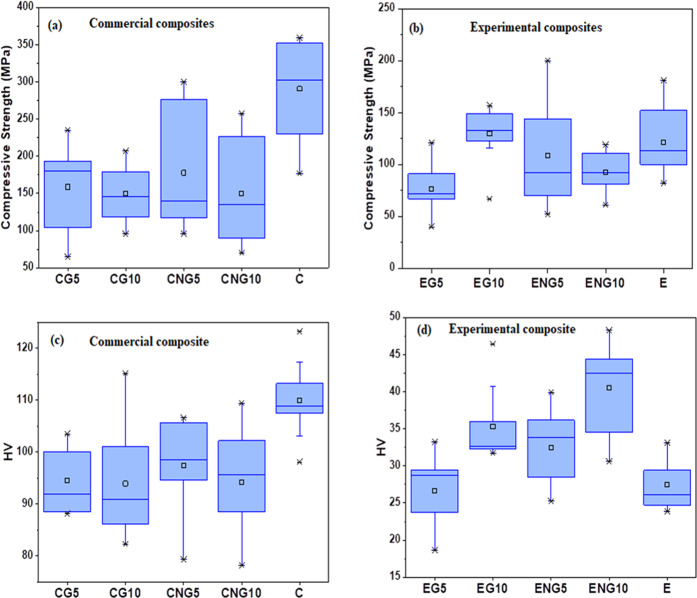
Mean CS and Vickers hardness values (**a** and **c**) of commercial composites and (**b** and **d**) experimental novel composites, respectively

The mean values of compressive strength of the composites are given in Fig. 4 a and b. The mean compressive strength values of all the experimental composite groups (EG5, EG10, ENG5, and ENG10) were less than all the commercial composite groups. This difference was statistically significant for all the groups (*p* < 0.05), except EG10 (134 ± 14 MPa) whose difference was not significant compared with CG10 (152 ± 34 MPa). Among the experimental composite groups, EG10 exhibited the highest compressive strength, however, nonsignificant (*p* > 0.05) within groups. The mean compressive strength value of this group was also higher than the negative control (E = 121 ± 32 MPa); however, a nonsignificant (*p* > 0.05) difference was observed. Other groups had lower compressive strength than their negative control, however, not significant statistically (*p* > 0.05).

The commercial composite groups (CG5, CG10, CNG5, and CNG10) exhibited less compressive strength than their negative control (C). This difference was statistically significant (*p* < 0.05). Among the commercial composite groups, CG5 (179 ± 45 MPa) exhibited the highest values followed by CNG5 (179 ± 76 MPa), CNG10 (178 ± 62 MPa), and CG10 (152 ± 32 MPa), respectively, however, the difference was nonsignificant (*p* > 0.05).

### Vickers hardness

The mean values of Vickers hardness (HV) for the commercial and experimental composite groups are given in Fig. 4 c and d, respectively. The mean HV values of the commercial composite groups (CG5, CG10, CNG5, and CNG10) were less than their negative control (C). This difference was statistically significant (*p* < 0.05). Among the groups (CG5, CG10, CNG5, and CNG10), the highest HV was exhibited by the CNG5 (97.43 ± 9.2) group followed by CG5 (94.49 ± 6.1), CNG10 (94.14 ± 10), and CG10 (93.93 ± 10), respectively. The difference among all these groups was nonsignificant (*p* > 0.05).

Among the experimental composite groups (EG5, EG10, ENG5, and ENG10), the mean HV value of the EG5 (26.63 ± 4.4) was nonsignificantly (*p* > 0.05) less than the control group E (27.45 ± 3.4). The groups (EG10, ENG5, and ENG10) exhibited mean HV value higher than control (E). This difference was statistically significant (*p* < 0.05) for ENG10 (40.5 ± 6) and nonsignificant (*p* > 0.05) for EG10 (34.63 ± 3.6) and ENG5 (32.5 ± 5.1).

### Abrasive wear resistance

#### Mass loss

**Fig. 5:**
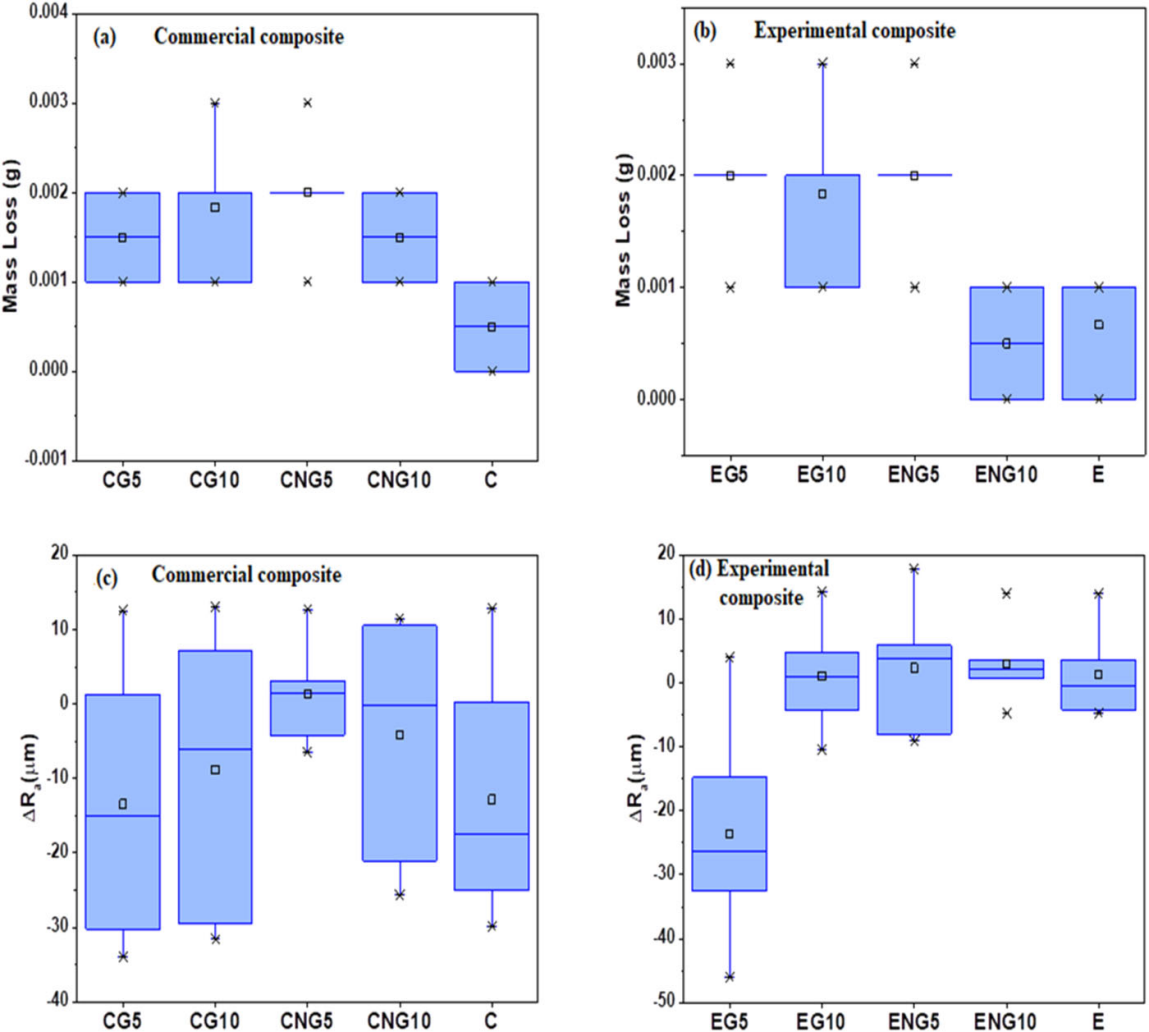
Mean mass loss and ΔRa values (**a** and **c**) Commercial composite groups (**b** and **d**) Experimental composite groups, respectively

The mean values of mass loss of all the groups of commercial composites (CG5, CG10, CNG5, CNG10, and C) and experimental composites (EG5, EG10, ENG5, ENG10, and E) were below 0.002 g (<2 mg), as shown in Fig. 5 a and b, respectively. The mean values of mass loss of commercial composites were higher than their control group C (0.14 ± 0.14 %), however, the difference was nonsignificant. The mass loss of CNG5 was significantly higher than the control. Among these groups, the least mean value of mass loss was 0.46 ± 0.14 %, exhibited by the composite CNG10 followed by CG5 (0.52 ± 0.20 %), CG10 (0.57 ± 0.25 %), and CNG5 (0.65 ± 0.20 %). The experimental composite groups exhibited the mean value of mass loss in an order from least to highest as ENG10 = E < EG10 < EG5 = ENG5, which was 0.19 ± 0.19%, 0.58 ± 0.24%, and 0.65 ± 0.21%, respectively.

#### Roughness average (ΔRa)

The mean Ra values and ΔRa of commercial composite groups (CG5, CG10, CNG5, CNG10, and C) are given in Table 2 and Fig. 5c, respectively. The mean Ra value of CNG5 increased, while mean Ra values of CG5, CG10, CNG10, and C decreased after simulated brushing and a decrease in value was the highest in CG5 followed by C, CG10, and CNG10, respectively. The difference among all the commercial composite groups was nonsignificant (*p* > 0.05).

**Table 2:** Pre and post-test roughness average of commercial and experimental novel composite groups

Group name	Roughness average (µm)	
	Ra_i_	Ra_f_
CG5	24.19 ± 16.37	10.73 ± 7.47
CG10	20.35 ± 13.43	11.51 ± 4.51
CNG5	8.49 ± 5.56	9.81 ± 5.74
CNG10	15.67 ± 13.59	11.63 ± 5.57
C	21.11 ± 16.37	8.29 ± 5.34
EG5	34.48 ± 11.87	9.53 ± 4.57
EG10	9.49 ± 3.77	10.51 ± 5.20
ENG5	12.20 ± 4.66	14.55 ± 7.51
ENG10	5.86 ± 3.27	11.33 ± 5.55
E	5.63 ± 2.56	6.83 ± 5.29

The mean Ra and ΔRa values of experimental composite groups are given in Table 2 and Fig. 5d, respectively. Among the experimental composite groups (EG5, EG10, ENG5, ENG10, and E), the mean Ra value of the group EG5 decreased while others’ increased. Among these groups, the only difference between mean Ra values of EG5 and ENG10 was significant (*p* < 0.05).

## Discussion

This study investigated and proved the claim that UDMA-grafted ZnO was incorporated into the dental composite. The grafting was successfully achieved by flow-synthesis method in the shortest possible time. The conventional way of silanization takes days [28], whereas, in the current study, the process of silanization was achieved in 1 h. During the silanization process, several factors can affect coupling efficiency, such as the nature of solvent, pH, and concentration of the coupling agent [26]. Moreover, MPS is hydrophobic in nature [32] that can restrict the interaction of physiological fluid with ZnO, subsequently it may reduce ion release. In this study, UDMA was grafted onto ZnO surface in situ. It is established that the interfacial interaction between the fillers and resin matrix plays an important role in determining the properties of the composites. The high surface area and surface energy of zinc nanoparticles can cause agglomeration; therefore, to improve the dispersion, it is necessary to alter the surface of ZnO nanoparticles [33]. UDMA contains the urethane group (H–N–C = O) along with C–H group. During the in situ reaction, it is expected that the hydroxyl group of ZnO was replaced with O–C–H of UDMA. This interaction is thought to be a hydrogen bond [34]. The FTIR spectrum confirmed the presence of UDMA on ZnO surface, whereby XRD pattern indicated that the morphology of ZnO was not changed during the reaction process. However, the intensity of the XRD was reduced due to the presence of UDMA. It is expected that grafting of urethane on ZnO surface led to formation of a monoblock system when incorporated as a filler in UDMA, *bis*-GMA, and TEGDMA-based resin matrices. Both *bis*-GMA and UDMA are large molecular weight monomers compared with TEGDMA, and during mixing of resin matrices, they underwent hydrogen bonding [35].

The prepared composites showed improved antibacterial activity with enhanced stability without decreasing compressive strength and Vickers hardness, and the maximum wear was within the limits specified by the American Dental Association for composite restorations. Antibacterial dental composites are clinically attractive as they can combat recurrent caries and minimize patients’ visits for treatment and their expenses as well [36].

The mechanism that explains the antibacterial activity of ZnO particles is the production of active oxygen species like H_2_O_2_ without needing of any extrinsic factors. The species produced by other metal oxides, like TiO_2_, inhibit growth of bacteria by this mechanism [37]. However, these species are produced in the presence of ultraviolet light [38]. Whereas, zinc ions (Zn^2+^) displace the magnesium ions in the bacterial cell wall and interfere with their enzyme system, resulting in bacterial inactivation [39].

In this study, the weight percentages of grafted and nongrafted ZnO in resin-matrix systems were 0%, 5%, and 10%, whereby UDMA was grafted on ZnO surfaces. The weight percentages of grafted and nongrafted ZnO nanoparticles were optimized in a pilot study and certain factors that can affect the composite’s properties were considered. One important factor, which can influence the photoactivated dental composite’s properties, is the differences in refractive indices of organic and inorganic components [40]. The difference in the refractive index of dimethacrylate resins [TEGDMA (1.46), UDMA (1.48), and *bis*-GMA (1.54)] and zinc oxide nanoparticles (2.02) could change the polymerization reaction of dental composites, and subsequently their physical and mechanical properties [41]. Therefore, it is important to consider the weight percentage of fillers in the resin-matrix system. The TGA confirmed that the amount of UDMA on ZnO surface was 28%.. UDMA-grafted ZnO exhibited greater antibacterial potential than the groups having bare ZnO. This might be attributed to less agglomeration and high dispersion of grafted ZnO in the resin matrix. It was also exhibited that antibacterial potential was significantly higher (*p* ≥ 0.05) after seven days as compared with days 1 and 3. The increase in antibacterial potential over time might be due to the continuous and increased release of Zn^2+^ ions, resulting in increased concentration of these ions and ultimately increased the antibacterial potential. The prolonged release of Zn^2+^ could be due to the nanostructure of the particles. It is reported that more active zinc atoms are present on the surface due to high surface-to-volume of the ZnO nanoparticles [42]. The other factor might be a change in pH during bacterial (S. *mutans*) incubation, which contains esterase. It is expected that with prolonged incubation (up to seven days), the pH reduced and also led to hydrolytic reaction. The release ratio of zinc ions increases as the pH decreases and the expected reaction is [43]

$${\rm ZnO} + 2{\rm H}^ + \to {\rm Zn}^{2 + } + {\rm H}_2{\rm O}$$

The ZnO nanoparticles are encapsulated in the resin matrix and prolonged immersion in media contributed to hydrolysis; however, this reaction is time-dependent as well. Therefore, more antibacterial activity was observed at day seven compared with days one and three. It is established that ZnO nanoparticles interact with the bacterial cell walls causing damage to the integrity of the bacterial cells, releasing Zn^2+^ ions mainly, and formation of reactive oxygen species (ROS) [43, 44]. The samples based on 10 wt.% concentration of UDMA-grafted ZnO exhibited better results than 5 wt.% with a possible attribution to more availability of ions and contact with bacteria. These findings are in accordance with previous studies [1, 45].

The dental composite having antibacterial potential should also possess satisfactory mechanical properties. The compressive strength of experimental novel composites was not significantly sacrificed after incorporation of UDMA-grafted or nongrafted ZnO. Even the compressive strength of the group containing 10 wt.% UDMA-grafted ZnO was higher than the control group due to an increase in filler content. After incorporation of 5 wt.% and 10 wt.% ZnO, filler content was much higher than the critical limit and might hinder the polymerization kinetics. The compressive strength of experimental novel composite groups was significantly less than commercial groups. This difference might be attributed to their lower filler content (40 wt.%) as compared with commercial composite groups (82 wt.%).

A similar trend was observed with Vickers micro-hardness results. There has been an established positive correlation between the surface hardness and inorganic filler content of dental resin composites [46, 47]. In the present study, among the experimental groups, the significantly high mean Vickers hardness was exhibited by the group containing the maximum filler content, which was ENG10. Other groups’ difference from control was not significant. Filler content of SiO_2_ was the same in all the groups, while ZnO was 10%, 6.6%, 5%, 3.3%, and 0% in ENG10, EG10, ENG5, EG5, and E, respectively. A decreasing trend was followed by the groups according to the filler content, revealing that Vickers hardness depends upon filler fraction in the resin composite.

All the composite groups, either experimental novel or commercial composites, exhibited the mean value of mass loss within the limit (below 0.002 g) according to the ISO/TR 14569. This mass loss in this study was in the range of 0.14 – 0.65% after 24,000 strokes. The mass loss within the limits indicated that the experimental novel and commercial composites were sufficiently resistant to abrasive wear necessary for long-term clinical stability. The average roughness value of the experimental novel composite groups including control increased after simulated tooth. The findings of the present study are in contrast to a previous study [48], reporting no influence of filler content.

## Conclusion

The hydrothermal technique was used to graft ZnO particles in situ. This study revealed that the novel UDMA-grafted ZnO-containing composite developed herein exhibited a greater antibacterial response against *S. mutans* as compared with those containing nongrafted ZnO. The antibacterial response of the composite containing 10 wt.% grafted ZnO was greater than that containing 5 wt.% grafted ZnO. There was an insignificant decrease in compressive strength and Vickers hardness of the experimental novel composite. The abrasive wear was within limits set by the American Dental Association. Regarding the comparison of the incorporation of UDMA-grafted ZnO into novel experimental and commercial composites, the latter showed antibacterial behavior similar to the former with a significant decrease in compressive strength and Vickers hardness. However, it is concluded that the group with 10% UDMA-grafted ZnO has shown the remarkable difference from other groups.
